# Silencing of a Wheat Ortholog of Glucan Synthase-Like Gene Reduced Resistance to *Blumeria graminis* f. sp. *tritici*

**DOI:** 10.3389/fpls.2021.800077

**Published:** 2021-12-23

**Authors:** Peng Cheng, Zihao Wang, Yanyan Ren, Pengfei Jin, Kangjie Ma, Qiang Li, Baotong Wang

**Affiliations:** State Key Laboratory of Crop Stress Biology for Arid Areas, College of Plant Protection, Northwest A&F University, Yangling, China

**Keywords:** wheat powdery mildew, BMSV-VIGS, disease resistance, callose, glucan synthase-like, *TaGSL22*

## Abstract

Wheat powdery mildew, caused by the obligate biotrophic ascomycete fungal pathogen *Blumeria graminis* f. sp. *tritici* (*Bgt*), is a major threat to wheat production worldwide. It is known that *Arabidopsis thaliana glucan synthase-like 5* (*AtGSL5*) improves the resistance of wheat to powdery mildew by increasing its anti-penetration abilities. However, the function of glucan synthase-like (*GSL*) orthologs in crop species remains largely unknown. In this study, *TaGSL22*, a novel functional ortholog of *AtGSL5*, was isolated as the only *Bgt*-induced *GSL* gene in wheat. Phylogenetic analysis indicated that *TaGSL22* was conserved within the group of Gramineae and showed a closer relationship to *GSL* orthologs from monocots than to those from dicots. The *TaGSL22* transcript was highest in the wheat leaves, followed by stems then roots. *TaGSL22* was localized in the cell membrane and cytoplasm of wheat protoplasts, as predicted by transmembrane structure analysis. In addition, expression of *TaGSL22* was induced by the plant hormones ethylene (ETH) and salicylic acid (SA), but down-regulated by jasmonate (JA) and abscisic acid (ABA). The transcript level of *TaGSL22* was up-regulated in the incompatible interaction between *Bgt* and wheat, whereas it remained relatively unchanged in the compatible interaction. Knocking down of *TaGSL22* by virus-induced gene silencing (VIGS) induced a higher infection type in the wheat–*Bgt* interaction. The *TaGSL22*-silenced plants exhibited reduced resistance to *Bgt*, accompanied by decreased callose accumulation. Our study shows a conserved function of *GSL* genes in plant immunity associated with penetration resistance, and it indicates that *TaGSL22* can be used to improve papilla composition and enhance resistance to wheat powdery mildew.

## Introduction

Wheat is one of the earliest cultivated crops in history. More than 10,000 years ago, humans began to plant *Triticum monococcum* for use as food (Araus et al., 2007). Today it remains the main source of calories and protein globally, and cultivating wheat varieties with high nutrition, yield, and resistance to biotic and abiotic stresses is key to ensuring food security (Mondal et al., 2016). Wheat powdery mildew is a fungal disease caused by *Blumeria graminis* f. sp. *tritici* (*Bgt*) (Belanger et al., 2003). The disease occurs in all wheat-growing regions in the world, and its distribution is very extensive. The reproduction of *Bgt* mainly depends on the conidia and ascospores of the pathogen, and the disease often occurs in a large area following the spread of airflow. Typically, wheat powdery mildew will cause a 5–19% yield loss of the crop, while in serious epidemic years that number may reach to more than 30%. As incidences of the disease increase, the safety of global wheat production is seriously threatened (Li et al., 2019). Planting disease-resistant wheat cultivars is the most effective and cost-effective strategy for the control of powdery mildew. At present, more than 60 loci of powdery mildew resistance genes have been identified, of which only a few have been cloned successfully, such as *Pm5e* and *Pm41* (Li et al., 2020; Xie et al., 2020). However, because most of these resistance genes are based on a gene-for-gene system and are race-specific, they are at risk of being overcome by new emerging races of the pathogen (McDonald and Linde, 2002). Genes that contribute to disease resistance with a broad spectrum are needed to provide durable resistance.

The cell wall of plants is the first barrier against the invasion of pathogens (Kacprzak et al., 2011). In the resistance of plants to pathogenic fungi and oomycetes, the plant cell wall will self-modify where the pathogens attempt to invade. This results in a complex structure known as a papilla, which is essentially a physical barrier created to slow down or even prevent the invasion of pathogens. The papilla contains a variety of chemical components with clear anti-microbial functions, such as thionins and phenolic compounds (McLusky et al., 1999). Callose, the most prominent component of the papillae, is likely indispensable in papillae disease resistance. The prevention of pathogen invasion has been observed to be associated with the deposit of relatively high amounts of callose at sites of attempted penetration, forming typical papillae (Voigt, 2016).

The glucan synthase-like (*GSL*) gene family exists in many plants and is responsible for the production of callose, which is present not only at sites of pathogen attack and fungal penetration, but also in the pollen cell wall and in callus plugs in wounds (Voigt et al., 2006). For example, Arabidopsis *thaliana glucan synthase-like 5* (*AtGSL5*) (also known as the *powdery mildew resistance gene 4*, *PMR4*) in *Arabidopsis thaliana* is involved in the formation of the callose wall that separates tetrads, making it essential for the formation of pollen (Enns et al., 2005). In addition, *AtGSL5* improves the resistance of *A. thaliana* to powdery mildew by increasing its anti-penetration abilities (Blümke et al., 2013). It was also found that CRISPR/Cas-9-targeted mutagenesis of the *PMR4* gene in tomato plants produced mutants that displayed a reduction (but not a complete loss) of susceptibility to the tomato powdery mildew pathogen *Oidium neolycopersici* (Martinez et al., 2020). The functional ortholog of *AtGSL5* in barley was identified as *HvGSL6*, whose down-regulation led to reduced glucan deposition and increased cell wall penetration by powdery mildew (Chowdhury et al., 2016). In wheat, eight different *TaGSL* genes were identified and ascertained to mediate the synthesis and regulation of callose under different environmental conditions in various tissues (Voigt et al., 2006). Functional analysis of members of the *GSL* gene family in wheat revealed that either *TaGSL8* or *TaGSL10* are involved in plant regeneration, while the *TaGSL3* and *TaGSL8* RNAi transgenic lines showed slightly reduced resistance against *Fusarium graminearum* (Rana et al., 2014). However, the *GSL* genes associated with papillary callose accumulation in common wheat have not been identified, and their organ- and tissue-specific expressions and involvement in disease resistance remain unknown.

A number of plant hormones play critical roles in the defense systems of plants (Shigenaga and Argueso, 2016). For example, salicylic acid (SA) is considered to be an important signaling molecule that induces an immune reaction in plants. It is involved in the hypersensitive response and the systemic acquired resistance response in a plant, both of which can regulate reactive oxygen species and antioxidants to improve plant resistance (Ryals et al., 1994). It has also been reported that the SA pathway in *A. thaliana* is negatively regulated by callose synthase, the loss of which can result in SA-dependent disease resistance to powdery mildew (Nishimura et al., 2003). Jasmonate (JA) is a plant hormone produced by the metabolism of unsaturated fatty acids, and it is involved in the regulation of plant resistance to pathogens, insects, and mechanical damage (Koo and Howe, 2009). In Arabidopsis, *AtGSL5* was found to negatively regulate JA and SA production or signaling to enhance disease resistance to powdery mildew, and the negative regulation of defense signaling by this gene could be independent of callose production (Wawrzynska et al., 2010). Ethylene (ETH) is a gaseous plant hormone derived from methionine, which is abundant in plants. The ETH response factor subfamily plays an important role in the establishment and regulation of defense systems of plants by balancing positive and negative transcriptional regulation (Bari and Jones, 2009). In fact, in most cases, these plant hormones are more effective when they act in relation to each other, making them indispensable members of the plant disease resistance response network. However, the regulatory function of these plant hormones in the defense of common wheat triggered by a *GSL* gene is not clear.

In the present study, the wheat ortholog gene *TaGSL22* of the Arabidopsis gene *AtGSL5* was identified in wheat. Transcript profiling of *TaGSL22* was analyzed in wheat seedlings inoculated with virulent and avirulent *Bgt* races, and in response to various plant hormones. Tissue-specific expression and subcellular localization of TaGSL22 were determined. The BMSV-VIGS technique was performed to demonstrate how *TaGSL22* was involved in callose regulation and disease resistance to *Bgt*. Taken together, our results indicated that the *TaGSL22* gene plays a role in callose synthesis and resistance to *Bgt* in wheat.

## Materials and Methods

### Plant Material and Pathogen Preparation

The common wheat variety *Asosan/8Cc*, which is highly resistant to *Bgt* E09 and susceptible to *Bgt* A13, was used throughout the study. The plants were placed under a transparent cover in a greenhouse with a temperature of 17°C, 75% humidity, and a 16 h photoperiod of light (18 μmol/m^2^/s). Inoculation with *Bgt* was carried out after about 7–10 days, when the wheat seedlings grew to one leaf (Zeng et al., 2010). Before inoculation, the seedlings were misted with water. Fresh conidia were then inoculated on the wheat leaves by the shaking powder method. About 7 days later, when the susceptible control “Jingshuang 16” reached infection type (IT) 4, the ITs of the tested materials were rated using a scale of 0–4 (Supplementary Table 1).

### Identification and Phylogenetic Analysis of *TaGSL22*

The protein sequence of *AtGSL5* in Arabidopsis was used to search the UniProt protein database for its ortholog gene in wheat. A 423 bp DNA sequence of *TaGSL22* was found in the linked NCBI database using the amino acid sequence of the TaGSL22 protein obtained in the UniProt protein database. Using this sequence, ortholog genes of *TaGSL22* in other plant species were identified by a BlastX search. A phylogenetic tree was constructed with sequences of the seven homologous genes obtained in BlastX using the molecular evolutionary genetic analysis software MEGA7. The signal peptide and transmembrane domain of the protein were predicted by online software TMHMM-2.0, and its subcellular localization was analyzed and predicted by online software cNLS Mapper.

### RNA Extraction and Quantitative Real-Time Polymerase Chain Reaction

Samples measuring about 100 mg were collected from leaves, stems, and roots at the seedling stage (7–10 days of growth), then frozen immediately in liquid nitrogen and stored at −80°C for RNA extraction using a plant RNA small extraction kit (Magen, Guangzhou, China). First-strand cDNA was synthesized in accordance with the instructions of the Hiscript III 1st Strand cDNA Synthesis Kit (+gDNA wiper) (Vazyme, Nanjing, China). For quantitative real-time polymerase chain reaction (qRT-PCR) analysis, ChamQ Universal SYBR qPCR Master Mix (Vazyme) was used on a Quant Studio 5 fluorescence quantitative instrument (Thermo Fisher, Waltham, MA, United States). Gene encoding wheat elongation factor (EF-1α) was used as the internal reference gene (Supplementary Table 1). After qRT-PCR analysis, the 2^–Δ Δ *CT*^ method was used to calculate the relative expression of the target gene (Livak and Schmittgen, 2001). The experiments consisted of three biological repeats and three technical repeats. The sequences of quantitative primers are shown in Supplementary Table 2.

### Subcellular Localization of TaGSL22::GFP Fusion Protein

The pJIT163-GFP vector was used to construct a *TaGSL22* expression vector (pJIT163-*TaGSL22*::GFP) for subcellular localization (Wang et al., 2013). Endonuclease *Bam*HI and *Xba*I were used to digest the pJIT163 plasmid, and the reaction was carried out on a Bio-Rad MyCycler PCR (Bio-Rad, Hercules, CA, United States) at 37°C for 4 h. After the reaction was complete, the product was recovered using a Gel Extraction Kit (Omega, Norcross, GA, United States). The *TaGSL22*, along with the recognition sequences and homologous arms, was amplified and ligated with the linear vector using a ClonExpress Ultra One Step Cloning Kit (Vazyme) with two CaMV 35S promoters. The ligated product was then transformed into *E. coli* DH5α and sequenced to confirm the successful construction of the *TaGSL22* expression vector. Wheat protoplasts were prepared using a wheat protoplast preparation and transformation kit (Coolaber, Beijing, China). The protoplasts were observed with a FV3000 confocal laser scanning microscope (Olympus Corp., Tokyo, Japan) using a GFP channel (Alexa Flour 488, green) and an SV2 channel (Alexa Flour 565, red).

### Transcript Profiling of *TaGSL22* Induced by Different Plant Hormones

The seedlings were treated with methyl jasmonate (MeJA) (0.1 mM), ETH (0.2 mM), abscisic acid (ABA) (0.2 mM), and SA (1 mM), respectively. Leaf samples were collected at 0, 0.5, 3, 6, 12, and 24 h after each treatment to conduct qRT-PCR analysis as described above. The mock control was treated with water. Data were normalized to the expression level of the wheat elongation factor TaEF-1α. The transcript level of genes in control plants at time 0 was standardized as 1. The qRT-PCR analysis for each respective experiment was repeated three times.

### Production of *TaGSL22*-Silenced Wheat Plants by Virus-Induced Gene Silencing

The viral vectors, including BMSV: α, BMSV: β, BMSV: γ, and BMSV: γ-phytoene desaturase gene (*PDS*), were donated by Professor Jun Guo at Northwest A&F University. The BSMV-VIGS system was performed as previously described (Yuan et al., 2011). *TaGSL22* was connected to BMSV: γ-*PDS* plasmid by homologous recombination to form γ-*TaGSL22* using a ClonExpress Ultra One Step Cloning Kit (Vazyme). BMSV: α and BMSV: γ were digested with endonuclease *Mlu*, BMSV: γ-*PDS* and BMSV: γ-*TaGSL22* were digested with *Bss*HII, BMSV: β was digested with *Spe*I, and the linearized vector was transcribed *in vitro* with an *in vitro* transcription kit (Promega, Madison, WI, United States). The transcripts of linearized plasmids α, β, and γ were mixed with appropriate amounts of 1 × FES buffer (0.1 M glycine, 0.06 M K_2_HPO_4_, 1% w/v tetrasodium pyrophosphate, 1% w/v bentonite, and 1% w/v celite, pH 8.5) (Pogue et al., 1998). When the seedlings reached the stage of two leaves and one heart, the second leaves were inoculated with the virus, and one pot of seedlings was kept uninoculated to serve as control. The wheat leaves were treated in darkness at 25°C for 24 h. After phenotypic observation, the virus-infected wheat seedlings were selected and inoculated with *Bgt* E09, then transferred to the growth chamber under the same conditions as described above. Samples were taken at 0, 48, and 120 h post infection (hpi) for qRT-PCR, and the silencing efficiency was measured. The primers used are listed in Supplementary Table 2.

### Histopathological Observation of the Powdery Mildew Pathogen in the *TaGSL22*-Silenced Plant

During virus-induced gene silencing (VIGS), histopathological observation was performed on samples taken at 24, 48, and 96 hpi with *Bgt* E09. The collected leaves were cut with scissors into 2 cm segments and placed in a decolorizing solution of anhydrous ethanol and glacial acetic acid for 2 days. The leaf segments were then transferred into a saturated chloral hydrate solution for transparency, and finally into a 50% glycerol solution. After wheat germ agglutinin (WGA) staining, the samples were then observed under an Olympus BX53 microscope (Olympus Corp., Tokyo, Japan). Representative colonies from the control and silenced groups were selected for imaging, and the colony areas, number of mycelial branches, and mycelium lengths were recorded.

### Microscopic Analysis of Callose Accumulation in the *TaGSL22*-Silenced Plant

During VIGS, freshly cut leaf tissues collected at 24 hpi with *Bgt* E09 were decolorized and washed twice with 50% (v/v) ethanol for 15 min, rinsed with water, then stained with aniline blue solution [67 mM K_2_HPO_4_, 0.05% (w/v) aniline blue]. The degree of callose deposition was determined in fields of 1 mm^2^ using a BX-51 microscope (Olympus Corp., Tokyo, Japan) (Xu et al., 2020). Representative pictures of the callose accumulation in the control and silenced groups, with scale bars measuring 100 μm, were selected for imaging. The average number of callose foci per field of view (1 mm^2^) was calculated from 30 fields.

### Statistical Analysis

The data processing system SPSS Statistics 25 (IBM, Armonk, NY, United States) was used for statistical analysis. The Student’s *t*-test method was used, wherein * and ** represented significant differences at *P* < 0.05 and *P* < 0.01, respectively. Relative gene quantification was calculated by the comparative ΔΔCT method. All experiments were performed and analyzed separately with three biological replicates.

## Results

### *TaGSL22* Gene Was Identified as a Wheat Ortholog of *Arabidopsis thaliana Glucan Synthase-Like 5*

To identify the putative ortholog of *AtGSL5* in common wheat, the UniProt protein database^1^ was searched using the amino acid sequence of the *AtGSL5* protein as the query sequence. A wheat ortholog gene with the highest sequence identity, *TaGSL22*, was extracted (UniProtKB: Q4JHU1).

The phylogenetic relationships of *GSL* genes in seven other plant species, *Aegilops tauschii*, *Hordeum vulgare*, *Setaria viridis*, *Zea mays*, *Oryza sativa*, *Gossypium hirsutum*, and *A. thaliana*, were investigated to identify their orthologs. A phylogenetic tree was then constructed using Mega 7 (Supplementary Figure 1A). It was inferred that the *GSL* gene was relatively conservative in Gramineae (in the same clade as *Hordeum* and *Aegilops*). The average hydrophobicity of the TaGSL22 protein was 0.289, indicating it was a hydrophobic protein. It was predicted that the TaGSL22 presented in cytoplasm and had transmembrane domains (Supplementary Figure 1B). According to the structure analysis, no signal peptide or nuclear localization were predicted.

### *TaGSL22* Expression Increased in the Resistance Reaction to *Bgt* E09

**FIGURE 1:**
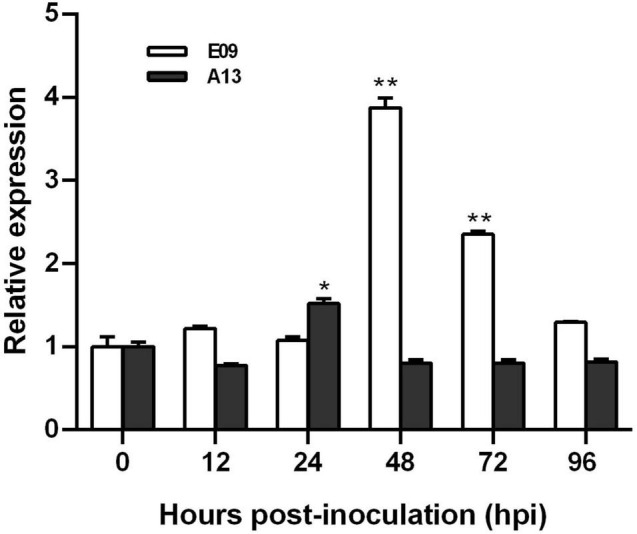
Transcript profiles of *TaGSL22* after inoculation with avirulent (E09) and virulent (A13) *Bgt* races. Relative gene quantification was calculated by the comparative ΔΔCT method. Data were normalized to the expression level of the wheat elongation factor TaEF-1α. * and ** indicate significant differences (*P* < 0.05 and *P* < 0.01) from the mock-inoculated control at 0 hpi using Student’s *t*-test. Error bars represent SEM of three biological replicates.

To characterize the expression pattern of *TaGSL22* in wheat after a fungal infection, its transcript profiles with races E09 (avirulent) and A13 (virulent) were examined using RT-qPCR (Figure 1). Expression levels were recorded in a time course at 12/24 h intervals across the first 96 h after inoculation. It was found that the expression of *TaGSL22* was increased at 48 hpi, and subsequently decreased with *Bgt* E09 infection. The peak at 48 hpi was about 3.8 times that of the control (0 hpi), indicating that gene expression was significantly induced. After 48 h, the expression of *TaGSL22* decreased slowly, but its value was always higher than during the first 24 h. In contrast, inoculation of *Bgt* A13 suppressed the overall expression of the *GSL* gene, except at 24 hpi when a 1.5 times expression was noted. These results indicated that *TaGSL22* was highly induced in the wheat powdery mildew resistance response, but not in the susceptible reaction.

### Expression of *TaGSL22* Was Highest in Leaves

**FIGURE 2:**
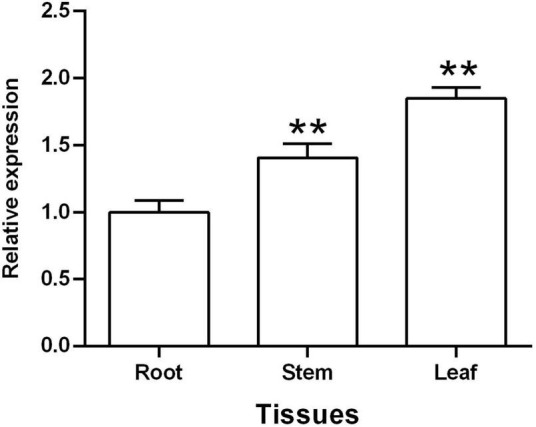
Tissue-specific expression of *TaGSL22*. Relative gene quantification was calculated by the comparative ΔΔCT method. Data were normalized to the expression level of the wheat elongation factor TaEF-1α. ** indicates a significant difference (*P* < 0.01) from the root by Student’s *t*-test. Error bars represent SEM of three biological replicates.

A qRT-PCR assay was used to assess the expression patterns of *TaGSL22* in different tissues, including root, stem, and leaf. The results showed that *TaGSL22* was detected in all tested plant tissues (Figure 2). *TaGSL22* had the highest expression in leaves and the lowest expression in roots. If the expression level in root tissues was defined as 1, then its expression in the stem and leaf tissues were 1.41 and 1.85, respectively.

### TaGSL22 Was Localized in the Cell Membrane and Cytoplasm of Wheat

**FIGURE 3:**
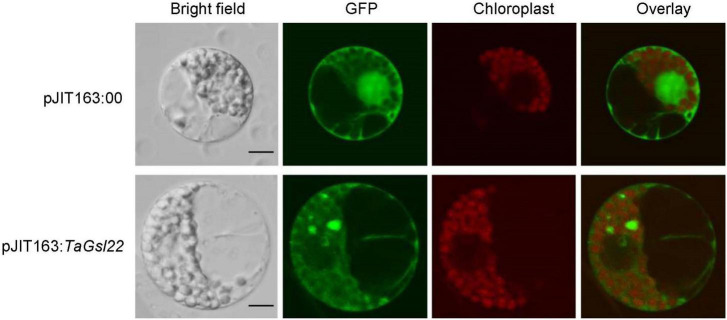
Subcellular localization of the TaGSL22 protein. pJIT163:00, pJIT163 empty plasmid; pJIT163:*TaGSL22*, pJIT163 recombinant plasmid with *TaGSL22*; overlay, superposition of green fluorescent protein (GFP) channel and chloroplast channel; bright field images show the equivalent field observed under white light. Scale bars: 10 μm.

To verify the subcellular localization predicted by the cNLS Mapper online software based on the amino acid sequence of the TaGSL22 protein, the pJIT163 empty plasmid (pJIT163:00) and pJIT163 recombinant plasmid with green fluorescent protein (GFP) fusion (pJIT163:*TaGSL22*) were transformed into wheat protoplasts. It was found that the protoplasts with pJIT163:00 emitted green fluorescence in the cell membrane, cytoplasm, and nucleus under the GFP channel, and under the SV2 channel, red fluorescence was observed only in the region where the organelles were located (Figure 3). In the protoplasts transformed with pJIT163:*TaGSL22*, the cell membrane and cytoplasm emitted green fluorescence under the GFP channel. These results suggested that the TaGSL22 protein was located in the cell membrane and cytoplasm, which was consistent with the initial prediction based on the amino acid sequence.

### *TaGSL22* Was Induced by Plant Hormones Ethylene and Salicylic Acid

**FIGURE 4:**
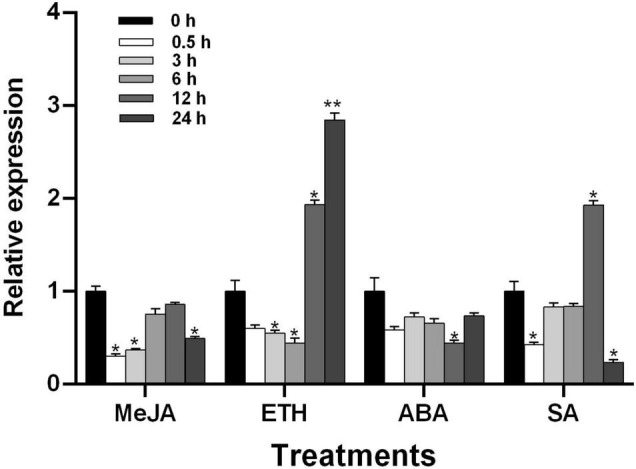
Transcript profiles of *TaGSL22* under different hormone treatments. MeJA, methyl jasmonate; ETH, ethylene; ABA, abscisic acid; SA, salicylic acid. The mock control was treated with water. Data were normalized to the expression level of the wheat elongation factor TaEF-1α. Relative gene quantification was calculated by the comparative ΔΔCT method. * and ** indicate a significant difference (*P* < 0.05 and *P* < 0.01) from the control at 0 hpt using Student’s *t*-test. Error bars represent SEM of three biological replicates.

To understand the regulatory effect of plant hormones on *TaGSL22* in wheat, the seedlings of variety *Asosan/8Cc* were treated with MeJA (0.1 mM), ETH (0.2 mM), ABA (0.2 mM), and SA (1 mM), respectively (Figure 4). After MeJA or ABA treatment, expression of *TaGSL22* was down-regulated. In contrast, the *TaGSL22* transcript level dropped to its minimum at 6 h post treatment (hpt) with ETH, then increased dramatically at 12 hpt until reaching its maximum at 24 hpt, at about 2.8 times that of the control (0 hpt). When treated with SA, expression of the gene decreased initially, then increased gradually to reach its maximum at 12 hpt, then decreased again. It was therefore inferred that *TaGSL22* may be involved in the ETH- and SA-mediated signaling pathways.

### Silencing of *TaGSL22* Reduced Resistance to Powdery Mildew in Wheat

**FIGURE 5:**
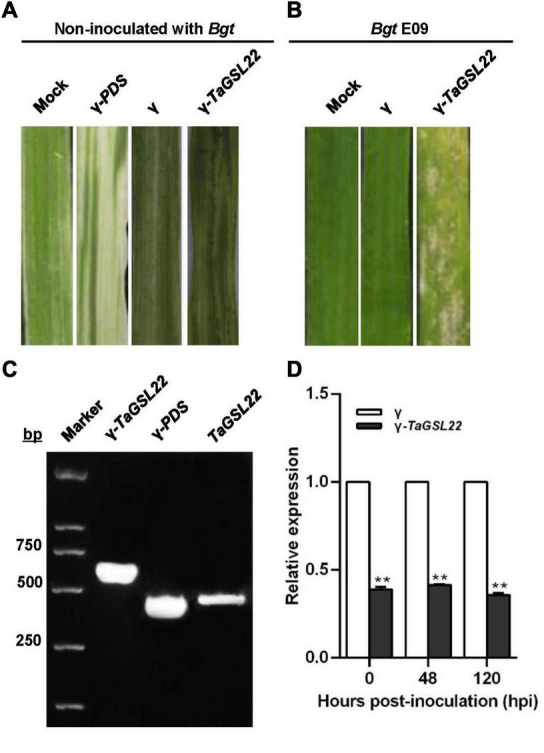
Functional assessment of *TaGSL22* in the wheat–*Bgt* interaction by gene silencing. **(A)** The leaf phenotype of successful virus infection; photographs were captured 12 days post-inoculation (dpi); mock: leaves inoculated with water; γ-*PDS*: transcripts of α, β, γ-*PDS* plasmids inoculated on leaves; γ: transcripts of α, β, γ plasmids inoculated on leaves; γ-*TaGSL22*: transcripts of α, β, γ-*TaGSL22* plasmids inoculated on leaves. **(B)** Disease phenotypes of the leaves pre-inoculated with BSMV: γ then the avirulent E09 *Bgt* race; mock: the leaves were only inoculated with *Bgt*. Photos were taken at 10 dpi. **(C)** PCR amplification of γ-*TaGSL22*, γ-*PDS*, and *TaGSL22* from corresponding leaves. **(D)** Assessment of *TaGSL22* silencing efficiency. Relative gene quantification was calculated by the comparative ΔΔCT method, and data were normalized to the expression level of the wheat elongation factor TaEF-1α. ** indicates a significant difference at *P* < 0.01 from the control group (γ) by Student’s *t*-test. Error bars represent SEM for three biological replicates.

To further assess the function of *TaGSL22* in wheat, the gene was silenced *via* a BSMV-based VIGS approach (Singh et al., 2012). Ten days after inoculation with γ-*PDS* as a control for VIGS, an efficient silencing of the *PDS* gene was indicated by the observation of photobleaching symptoms on the leaves (Figure 5A). The leaves inoculated with both the γ virus and the γ-*TaGSL22* virus exhibited chlorotic stripes, and no symptoms were observed on the leaves without virus inoculation. To evaluate the role of *TaGSL22* in resistance against the powdery mildew pathogen *Bgt*, the fourth leaf with evident virus virulence was inoculated with *Bgt* E09 and the disease IT was scored at 10 dpi. The gene-silenced group was found to have significantly more spores on its leaves than on leaves in the control groups (Figure 5B).

The involvement of *TaGSL22* in the plant resistance reaction was verified by PCR amplification of γ-*TaGSL22*, γ-*PDS*, and *TaGSL22* from corresponding leaves. qRT-PCR analysis was carried out to determine whether the gene was effectively silenced at the transcript level (Figure 5C). At three time points, the endogenous *TaGSL22* transcript levels were significantly reduced in leaves inoculated with the γ-*TaGSL22* virus compared with those in control plants (Figure 5D). The results indicated that the wheat resistance to *Bgt* was reduced after down-regulation of *TaGSL22*.

### Down-Regulation of *TaGSL22* Led to Significantly More Virulent Fungal Infections

**FIGURE 6:**
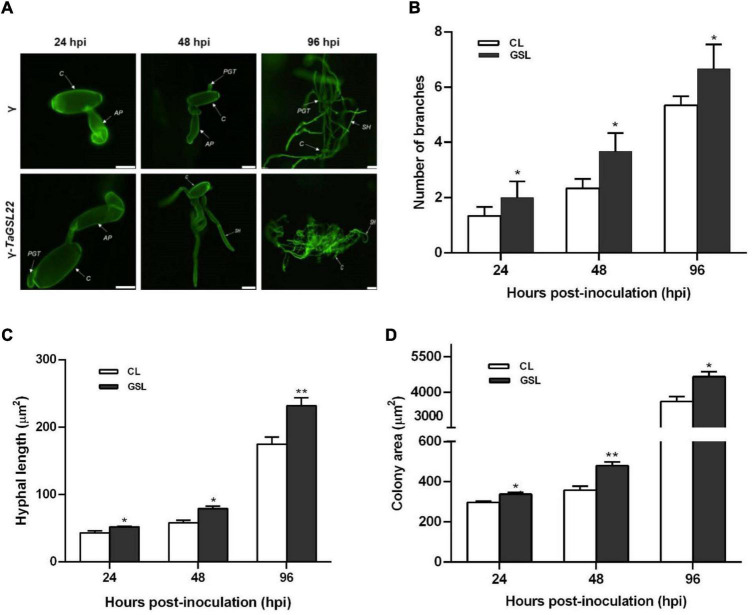
**(A)** Histopathological observation of *Bgt* infection on leaves after gene silencing. Samples of control plants (γ) and the gene-silenced plants (γ-*TaGSL22*) were taken at 24, 48, and 96 hpi. The fungal structures were stained with wheat germ agglutinin (WGA). C, conidia; AP, appressorium; PGT, primary germ tube; SH, secondary hyphae. Scale bars: 5 μm. **(B)** Number of branches, **(C)** hyphal length, and **(D)** colony area of *Bgt* after gene silencing. The leave samples were collected at 24, 48, and 96 hpi, and all results were obtained from 50 infection sites. CL, control leaf; *GSL*, gene-silenced leaf. Error bars represent SEM of three biological replicates. * and ** represent significant differences at *P* < 0.05 and *P* < 0.01, respectively, from the control by Student’s *t*-test.

To further clarify the role of *TaGSL22* during the fungal colonization on leaves, samples were taken at three time points and observed under a fluorescence microscope (Figure 6A). At 24 hpi, the appressoria were two times longer in the *TaGSL22* down-regulated leaves (γ-*TaGSL22*) compared to those in the control plants (γ). Secondary hypha were observed at 48 hpi in the *TaGSL22*-silenced leaves, while in the control plants, the primary germ tube was only just germinated from the conidium. At 96 hpi, the hypha mass was significantly larger in the gene-silenced plants than in the control plants. The leaves of the gene-silenced group displayed a significant increase in the number of mycelia branches and mycelia lengths compared to those in the control group, and the powdery mildew colony areas were also larger (Figures 6B–D). Over time, all three measured parameters increased dramatically at the histological level. A considerably faster growth of the fungus in the *TaGSL22*-silenced plants suggested that down-regulation of *TaGSL22* increased the *Bgt* fungal infection.

### Silencing of *TaGSL22* Resulted in Reduced Levels of Callose

**FIGURE 7:**
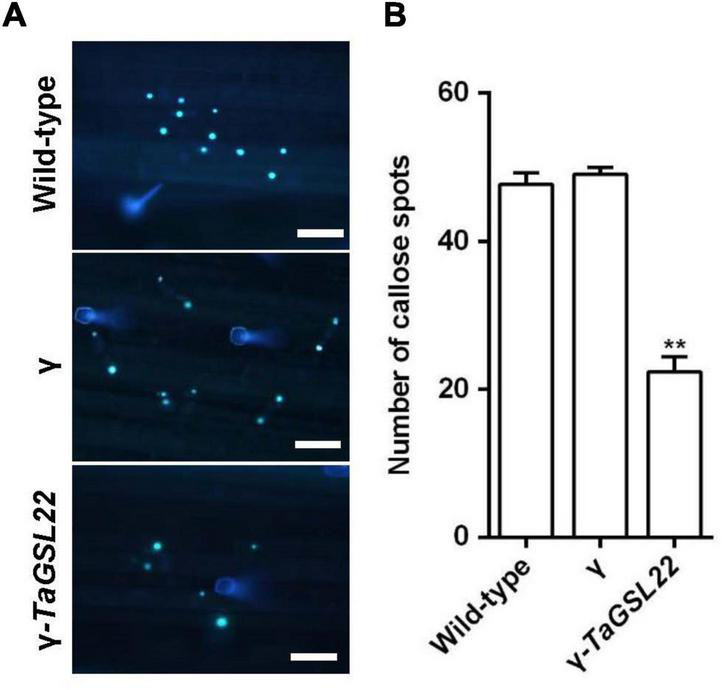
Down-regulation of *TaGSL22* expression reduced the callose deposition in host plant. **(A)** Representative wheat leaves stained with aniline blue for callose deposition (bar = 100 μm). Pictures of control plants (wild-type and γ) and the gene-silenced plants (γ-*TaGSL22*) were taken at 24 hpi with *Bgt* avirulent race E09 at ×10 magnification with an Olympus BX-51 microscope. **(B)** The average number of callose foci per field of view (1 mm^2^) was calculated from 30 fields for each sample. Error bars represent SEM of three biological replicates. ** represent significant differences at *P* < 0.01 (Student’s *t*-test).

In order to determine whether the silencing of the *TaGSL22* gene affected the deposition of callose in epidermal cells during *Bgt* infection, infected wheat leaves were stained with aniline blue to examine callose accumulation (Figure 7A). Samples of control plants (wild-type and γ) and the gene-silenced plants (γ-*TaGSL22*) were collected at 24 hpi with *Bgt* E09 and stained, then microscopic observation was carried out. It was observed that the fluorescent signals detected in the gene-silenced plants were significantly reduced compared to signals in the control plants, whereas the wild-type and empty vector plants showed similar intensities of callose accumulation signals (Figure 7B).

## Discussion

### Callose Synthase Glucan Synthase-Likes Play Different Roles in Various Plant Species and Tissues

Callose, a β-1,3-glucan, plays critical regulatory roles in plants, such as in sieve tube metabolism and gametophyte development (Legentil et al., 2015; Shi et al., 2015). It has long been observed that the deposition of relatively high amounts of callose and cellulose at sites of attempted penetration induces the formation of effective papillae, preventing the invasion of pathogens (Voigt, 2016). The most prominent cell wall polymer of papillae is β-1,3-glucan callose (Mangin, 1895). It has been reported that callose can enhance the resistance of melon to aphids (Shinoda, 1993), and early callose deposition increased the resistance of Arabidopsis to powdery mildew (Ellinger et al., 2013). Furthermore, overexpression of the Arabidopsis callose synthase gene *AtGSL5* in barley induced penetration resistance to the barley powdery mildew pathogen *Blumeria graminis* f. sp. *hordei* (*Bgh*) (Blümke et al., 2013). Silencing of *HvGSL6*, encoding the barley callose synthase that falls into the *AtGSL5* clade, also caused a decline in callose accumulation in powdery mildew-induced papillae and resulted in an increased penetration rate of *Bgh* (Chowdhury et al., 2016).

To date, a number of *TaGSL* genes have been identified in different wheat tissues with various regulatory functions under different environmental conditions (Voigt et al., 2006). *TaGSL8* or *TaGSL10* are involved in plant regeneration, and the knocking down of *TaGSL3* and *TaGSL8* slightly reduced the resistance against *F. graminearum* (Rana et al., 2014). In the present study, phylogenetic analysis revealed that *TaGSL22* had the closest sequence similarity with *AtGSL5*. Transcript profile analysis at different time points revealed a significant up-regulation of the *TaGSL22* gene following infection by *Bgt*. As *AtGSL5* was the only *GSL* induced during pathogen infection (Dong et al., 2008), our results suggested that *TaGSL22* was likely the ortholog of *AtGSL5*. The tissue-specific expression analysis of *TaGSL22* showed that its expression was highest in the leaves compared to the roots and stems, which is consistent with the finding by Voigt et al. (2006). Silencing of *TaGSL22* reduced the accumulation of callose significantly and increased the *Bgt* growth rate. Our study is the first report on the *GSL* genes associated with papillary callose accumulation and powdery mildew resistance in common wheat.

### Pathogen-Induced Callose Synthase in Poaceae May Be Involved in Different Plant Defense Regulating Pathways Than Dicots

Unlike in barley and wheat, it was found that silencing the pathogen-induced callose synthase *AtGSL5* in Arabidopsis results in a significant reduction of callose deposition, leading to an unexpected increase in fungal resistance (Jacobs et al., 2003). Similarly, silencing of the callose synthase gene *SlPMR4*, the ortholog of *AtGSL5* in tomato, resulted in increased resistance to the adapted powdery mildew pathogen *O. neolycopersici* (Huibers et al., 2013). Interestingly, the increased resistance in Arabidopsis has previously been explained to result from a hyperactivation of the SA-dependent defense pathway due to the loss of callose (Nishimura et al., 2003). It has also been suggested that SA may be involved in the regulation of stress response genes, since similar results were found in a study of induced plant defense responses against chewing insects in Arabidopsis (Stotz et al., 2000). Although reciprocal phenomena were observed from the down-regulation of *GSL* in Arabidopsis and barley, the function of callose deposition was verified by the overexpression of *AtGSL5* in barley and Arabidopsis, resulting in complete penetration resistance to *Bgh* and *Golovinomyces cichoracearum*, respectively (Blümke et al., 2013; Ellinger et al., 2013). Combining this result with the fact that silencing the *HvGSL6* gene leads to a loss-of-resistance phenotype that is not clouded by an off-target increase in the SA-dependent defense pathway, Chowdhury et al. (2016) proposed that the pathogen-induced callose synthase observed in barley may not be involved in regulating other plant defense pathways, as compared to observations in the dicotyledonous plants Arabidopsis and tomato.

In the present study, it was indicated that the expression of *TaGSL22* increased at 12 hpt with ETH and SA, respectively. Interestingly, reciprocal trends were observed at 24 hpt. While the transcript level of TaGSL22 kept increasing under ETH treatment, the *TaGSL22* expression was suppressed by SA. Considering the loss of callose induced SA-dependent defense pathways in Arabidopsis, further investigation is needed to understand the relationship between *GSL* regulation and SA-dependent defense pathways in wheat and barley.

### Utilization of *TaGSL22* in Wheat Resistance Breeding

The nucleotide sequence of *TaGSL22* had the highest similarity with those of *A. tauschii* and *H. vulgare*, indicating that the gene is relatively conservative in Gramineae plants and evolves slowly. This gives new opportunities for investigating orthologous genes in relative species and provides new targets for increasing crop resistance against penetration. Our study identified a novel wheat ortholog gene, *TaGSL22*, that may contribute to improved resistance in wheat breeding. In future studies, we plan to search the wheat whole-genome resequencing database for any natural variations in *TaGSL22* that can be identified for breeding applications. Molecular markers can be developed and tested in a natural wheat accession to verify the association between *Bgt* resistance and the *TaGSL22* allele. The identified polymorphic markers could then be used for marker-assisted selection for introgression of *TaGSL22* in wheat resistance breeding.

It is noteworthy that although *HvGSL6* has been identified in barley and its RNAi transgenic lines resulted in increased penetration by *Bgh* (Chowdhury et al., 2016), studies on the overexpression of *HvGSL6* have not been reported. We are working on developing *TaGSL22* overexpression wheat lines to further investigate its positive regulation of resistance to wheat powdery mildew. However, transgenic crops are not widely accepted for agricultural production. Recently, A donor-DNA-free CRISPR/Cas-based approach was reported to increase expression of target genes by creating structural variations in rice (Lu et al., 2021). This cutting-edge technology knocks out the sequence between the target gene and the promotor of a neighboring high-expressed gene, resulting in gene knock-up by ligating the high-expressed promotor with the coding region of the target gene. It is believed that this advanced gene knock-up technology sheds light on the potential applications of an identified resistance gene in crop resistance enhancement without introducing alien DNA residues by plant transformation.

## Data Availability Statement

The datasets presented in this study can be found in online repositories. The names of the repository/repositories and accession number(s) can be found in the article/Supplementary Material.

## Author Contributions

PC and ZW designed the experiments and wrote the manuscript. ZW, YR, and PC performed the experiments. ZW, PC, PJ, KM, and QL analyzed the data. PC acquired the funding. BW supervised the project. All authors read and approved the final manuscript.

## Conflict of Interest

The authors declare that the research was conducted in the absence of any commercial or financial relationships that could be construed as a potential conflict of interest.

## Publisher’s Note

All claims expressed in this article are solely those of the authors and do not necessarily represent those of their affiliated organizations, or those of the publisher, the editors and the reviewers. Any product that may be evaluated in this article, or claim that may be made by its manufacturer, is not guaranteed or endorsed by the publisher.

## Footnotes

1
https://www.uniprot.org/

## References

[B1] Araus J. L. Ferrio J. P. Buxo R. Voltas J. (2007). The historical perspective of dryland agriculture: Lessons learned from 10,000 years of wheat cultivation. *J. Exp. Bot.* 58 131–145. 10.1093/jxb/erl133 17050642

[B2] Bari R. Jones J. D. (2009). Role of plant hormones in plant defence responses. *Plant. Mol. Biol.* 69 473–488. 10.1007/s11103-008-9435-0 19083153

[B3] Belanger R. R. Benhamou N. Menzies J. G. (2003). Cytological Evidence of an Active Role of Silicon in Wheat Resistance to Powdery Mildew (*Blumeria graminis* f. sp. *tritici*). *Phytopathology* 93 402–412. 10.1094/PHYTO.2003.93.4.402 18944354

[B4] Blümke A. Somerville S. C. Voigt C. A. (2013). Transient expression of the *Arabidopsis thaliana* callose synthase PMR4 increases penetration resistance to powdery mildew in barley. *Adv. Biosci. Biotechnol.* 4 810–813. 10.4236/abb.2013.48106

[B5] Chowdhury J. Schober M. S. Shirley N. J. Singh R. R. Jacobs A. K. Douchkov D. (2016). Down-regulation of the glucan synthase-like 6 gene (*HvGsl6*) in barley leads to decreased callose accumulation and increased cell wall penetration by *Blumeria graminis* f. sp. *hordei*. *New Phytol.* 212 434–443. 10.1111/nph.14086 27364233

[B6] Dong X. Hong Z. Chatterjee J. Kim S. Verma D. P. S. (2008). Expression of callose synthase genes and its connection with Npr1 signaling pathway during pathogen infection. *Planta* 229 87–98. 10.1007/s00425-008-0812-3 18807070

[B7] Ellinger D. Naumann M. Falter C. Zwikowics C. Jamrow T. Manisseri C. (2013). Elevated early callose deposition results in complete penetration resistance to powdery mildew in Arabidopsis. *Plant. Physiol.* 161 1433–1444. 10.1104/pp.112.211011 23335625 PMC3585607

[B8] Enns L. C. Kanaoka M. M. Torii K. U. Comai L. Okada K. Cleland R. E. (2005). Two callose synthases, GSL1 and GSL5, play an essential and redundant role in plant and pollen development and in fertility. *Plant. Mol. Biol.* 58 333–349. 10.1007/s11103-005-4526-7 16021399

[B9] Huibers R. P. Loonen A. E. Gao D. Van den Ackerveken G. Visser R. G. Bai Y. (2013). Powdery mildew resistance in tomato by impairment of *SlPMR4* and *SlDMR1*. *PLoS One* 8:e67467. 10.1371/journal.pone.0067467 23818978 PMC3688610

[B10] Jacobs A. K. Lipka V. Burton R. A. Panstruga R. Strizhov N. Schulze-Lefert P. (2003). An Arabidopsis callose synthase, GSL5, is required for wound and papillary callose formation. *Plant Cell* 15 2503–2515. 10.1105/tpc.016097 14555698 PMC280557

[B11] Kacprzak P. Macioszek V. K. Kononowicz A. K. (2011). Induced systemic resistance (isr) in the protection of plants against pathogenic fungi. *Postepy. Biol. Komorki.* 38 129–142.

[B12] Koo A. J. Howe G. A. (2009). The wound hormone jasmonate. *Phytochemistry* 70 1571–1580. 10.1016/j.phytochem.2009.07.018 19695649 PMC2784233

[B13] Legentil L. Paris F. Ballet C. Trouvelot S. Daire X. Vetvicka V. (2015). Molecular interactions of β-(1→ 3)-glucans with their receptors. *Molecules* 20 9745–9766. 10.3390/molecules20069745 26023937 PMC6272582

[B14] Li G. Cowger C. Wang X. Carver B. F. Xu X. (2019). Characterization of *Pm65*, a new powdery mildew resistance gene on chromosome 2AL of a facultative wheat cultivar. *Theor. Appl. Genet.* 132 2625–2632. 10.1007/s00122-019-03377-2 31214740

[B15] Li M. M. Dong L. L. Li B. B. Wang Z. Z. Xie J. Z. Qiu D. (2020). A CNL protein in wild emmer wheat confers powdery mildew resistance. *New Phytol.* 228 1027–1037. 10.1111/nph.16761 32583535

[B16] Livak K. J. Schmittgen T. D. (2001). Analysis of Relative Gene Expression Data Using Real-Time Quantitative PCR and the 2^–Δ Δ *CT*^ Method. *Methods* 25 402–408. 10.1006/meth.2001.1262 11846609

[B17] Lu Y. Wang J. Chen B. (2021). A donor-DNA-free CRISPR/Cas-based approach to gene knock-up in rice. *Nat. Plants* 7 1445–1452. 10.1038/s41477-021-01019-4 34782773

[B18] Mangin L. (1895). Recherches sur les Péronosporées. *Bull. Société d’Hist. Nat. d’Autun* 8 55–108.

[B19] Martinez M. I. S. Bracuto V. Koseoglou E. Appiano M. Jacobsen E. Visser R. G. F. (2020). CRISPR/Cas9-targeted mutagenesis of the tomato susceptibility gene *PMR4* for resistance against powdery mildew. *BMC Plant. Biol.* 20:284–287. 10.1186/s12870-020-02497-y 32560695 PMC7304142

[B20] McDonald B. A. Linde C. (2002). The population genetics of plant pathogens and breeding strategies for durable resistance. *Euphytica* 124 163–180. 10.1023/A:1015678432355

[B21] McLusky S. R. Bennett M. H. Beale M. H. Lewis M. J. Gaskin P. Mansfield J. W. (1999). Cell wall alterations and localized accumulation of feruloyl-3’-methoxytyramine in onion epidermis at sites of attempted penetration by Botrytis allii are associated with actin polarisation, peroxidase activity and suppression of flavonoid biosynthesis. *Plant. J.* 17 523–534. 10.1046/j.1365-313X.1999.00403.x

[B22] Mondal S. Singh R. P. Mason E. R. Huerta-Espino J. Autrique E. Joshi A. K. (2016). Grain yield, adaptation and progress in breeding for early-maturing and heat-tolerant wheat lines in South Asia. *Field Crops Res.* 192 78–85. 10.1016/j.fcr.2016.04.017 27307654 PMC4892352

[B23] Nishimura M. T. Stein M. Hou B. H. Vogel J. P. Edwards H. Somerville S. C. (2003). Loss of a callose synthase results in salicylic acid-dependent disease resistance. *Science* 301 969–972. 10.1126/science.1086716 12920300

[B24] Pogue G. P. Lindbo J. A. Dawson W. O. Turpen T. H. (1998). “Tobamovirus transient expression vectors: tools for plant biology and high level expression of foreign proteins in plants,” in *Plant Molecular Biology Manual*, eds Gelvin S. B. Schilperoot R. A. (Dordrecht: Kluwer Academic Publishers), 1–27. 10.1385/0-89603-321-x:1

[B25] Rana I. A. Salomon S. Schafer W. Becker D. (2014). Downregulation of *Glucan Synthase-Like* (*TaGSL*) genes in wheat leads to inhibition of transgenic plant regeneration. *In Vitro Cell. Dev. Biol. Plant.* 50 696–706. 10.1007/s11627-014-9636-y

[B26] Ryals J. Uknes S. Ward E. (1994). Systemic Acquired-Resistance. *Plant. Physiol.* 104 1109–1112. 10.4161/psb.1.4.3221 12232151 PMC159270

[B27] Shi X. Sun X. Zhang Z. Feng D. Zhang Q. Han L. (2015). GLUCAN SYNTHASE-LIKE 5 (*GSL5*) plays an essential role in male fertility by regulating callose metabolism during microsporogenesis in rice. *Plant. Cell Physiol.* 56 497–509. 10.1093/pcp/pcu193 25520407

[B28] Shigenaga A. M. Argueso C. T. (2016). No hormone to rule them all: Interactions of plant hormones during the responses of plants to pathogens. *Semin. Cell Dev. Biol.* 56 174–189. 10.1016/j.semcdb.2016.06.005 27312082

[B29] Shinoda T. (1993). Callose reaction induced in melon leaves by feeding of melon aphid, *Aphis gossypii* Glover as possible aphid-resistant factor. *Jpn. J. Appl. Entomol. Zool.* 37 145–152. 10.1303/jjaez.37.145 33922110

[B30] Singh A. Liang Y. C. Kumar P. Jiang C. Z. Reid M. S. (2012). Co-silencing of the Mirabilis antiviral protein (MAP) permits virus-induced gene silencing (VIGS) of other genes in Four O’Clock plants (*Mirabilis jalapa*). *J. Horticult. Sci. Biotechnol.* 87 334–340. 10.1080/14620316.2012.11512873

[B31] Stotz H. U. Pittendrigh B. R. Kroymann J. Weniger K. Fritsche J. Bauke A. (2000). Induced plant defense responses against chewing insects. Ethylene signaling reduces resistance of Arabidopsis against Egyptian cotton worm but not diamondback moth. *Plant. Physiol.* 124 1007–1018. 10.1104/pp.124.3.1007 11080278 PMC59200

[B32] Voigt C. A. (2016). Cellulose/callose glucan networks: The key to powdery mildew resistance in plants? *New Phytol.* 212 303–305. 10.1111/nph.14198 27641960

[B33] Voigt C. A. Schafer W. Salomon S. (2006). A comprehensive view on organ-specific callose synthesis in wheat (*Triticum aestivum* L.): Glucan synthase-like gene expression, callose synthase activity, callose quantification and deposition. *Plant. Physiol. Biochem.* 44 242–247. 10.1016/j.plaphy.2006.05.001 16777426

[B34] Wang X. Bai J. Liu H. Sun Y. Shi X. Ren Z. (2013). Overexpression of a maize transcription factor ZmPHR1 improves shoot inorganic phosphate content and growth of Arabidopsis under low-phosphate conditions. *Plant. Mol. Biol. Rep.* 31 665–677. 10.1007/s11105-012-0534-3

[B35] Wawrzynska A. Rodibaugh N. L. Innes R. W. (2010). Synergistic activation of defense responses in Arabidopsis by simultaneous loss of the GSL5 callose synthase and the EDR1 protein kinase. *Mol. Plant. Microbe Interact.* 23 578–584. 10.1094/MPMI-23-5-0578 20367466 PMC3290096

[B36] Xie J. Guo G. Wang Y. Hu T. Wang L. Li J. (2020). A rare single nucleotide variant in *Pm5e* confers powdery mildew resistance in common wheat. *New Phytol.* 228 1011–1026. 10.1111/nph.16762 32569398

[B37] Xu Q. Tang C. L. Wang L. K. Zhao C. C. Kang Z. S. Wang X. J. (2020). Haustoria - arsenals during the interaction between wheat and *Puccinia striiformis* f. sp. *tritici*. *Mol. Plant Pathol.* 21 83–94. 31774224 10.1111/mpp.12882 PMC6913192

[B38] Yuan C. Li C. Yan L. Jackson A. O. Liu Z. Han C. (2011). A high throughput barley stripe mosaic virus vector for virus induced gene silencing in monocots and dicots. *PLoS One* 6:e26468. 10.1371/journal.pone.0026468 22031834 PMC3198768

[B39] Zeng X. W. Luo Y. Zheng Y. M. Duan X. Y. Zhou Y. L. (2010). Detection of latent infection of wheat leaves caused by *Blumeria graminis* f. sp. *tritici* using nested PCR. *J. Phytopathol.* 158 227–235. 10.1111/j.1439-0434.2009.01594.x

