# Supplementary material for: Silencing of a Wheat Ortholog of Glucan Synthase-Like Gene Reduced Resistance to Blumeria graminis f. sp. tritici
Source: Front Plant Sci. 2021 Dec 23;12:800077. doi: 10.3389/fpls.2021.800077 (PMC8735228; doi:10.3389/fpls.2021.800077)
Supplement: Supplementary file 1 [file Data_Sheet_1.docx]

**SUPPLEMENT**

**Contents**

**Tables S1-S2**

**Table S1**. Evaluation and description of infection types, symptoms and resistance of wheat powdery mildew at seedling stage.

| Infection types | Symptom description | Resistance evaluation |
| --- | --- | --- |
| 0 | Plant without disease spot. | Immunity |
| 0; | Necrotic reaction, there are dead spots on the leaves. | Near immunity |
| 1 | The diameter of scab is less than 1 mm, the mycelium is thin and transparent. | High resistance |
| 2 | The diameter of scab is less than 1 mm, opaque. | Moderate resistance |
| 3 | The diameter of the scab is larger than 1mm, the mycelium is thicker, and the plaque is more but not connected. | Moderate susceptibility |
| 4 | The diameter of the scab is larger than 1mm, the mycelium is thicker, and the plaque is more and connected. | High susceptibility |

**Table S2**. Primer sequences and amplicon lengths in the study. *EF-1α*: internal reference gene; VIGS-q: The primer used to detect the gene silencing efficiency in VIGS; 163 and 163-*GSL22*: The primers used to construct and detect vectors in subcellular localization; γ and γ-*GSL22*: The primers used to construct and detect vectors in VIGS; q-*GSL22*: primers for qRT-PCR.

| Primer | | Primer sequence 5'-3' | Product length (bp) | |
| --- | --- | --- | --- | --- |
| *EF-1α*-F | | TGGTGTCATCAAGCCTGGTATGGT | 146 | |
| *EF-1α*-R | | ACTCATGGTGCATCTCAACGGACT |  |  |
| VIGS-q-F | | TACATCCTGCTGACGCTT | 155 | |
| VIGS-q-R | | TTCACTGAGATTCCACCCT |  |  |
| 163-F | | ATTTCATTTGGAGAGGAC | 201 | |
| 163-R | | TCAGGGTCAGCTTGCCG |  |  |
| 163-*GSL22*-F | | AGGTCGACGATATCTCTAGAATGTTTGAAGCCAAGGTTGCTAG | 510 | |
| 163-*GSL22*-R | | CCCTTGCTCACCATGGATCCCTCGGCGAATTTCTTGTGCT |  |  |
| γ-F | | AAAGTGAGGTTAACGCAATACG | 415 | |
| γ-R | | TCAGGCATCGTTTTCAAGTT |  |  |
| γ-*GSL22*-F | | TCCGTTGCTAGCTGAGCGGCCGCCCGGATGCTCTCGTTTT | 722 | |
| γ-*GSL22*-R | | TTTTTAGCTAGCTGATTAATTAATCGGCGAATTTCTTGTG |  |  |
| *GSL22*-F | | CCGGATGCTCTCGTTTT | 423 | |
| *GSL22*-R | | TCGGCGAATTTCTTGTG |  |  |
| q-*GSL22*-F | GTCTGGGGGCGATTTTA | | 118 |  |
| q-*GSL22*-R | GCTGTATGACGAACTGCTGA | |  |  |

**Figure S1**

**
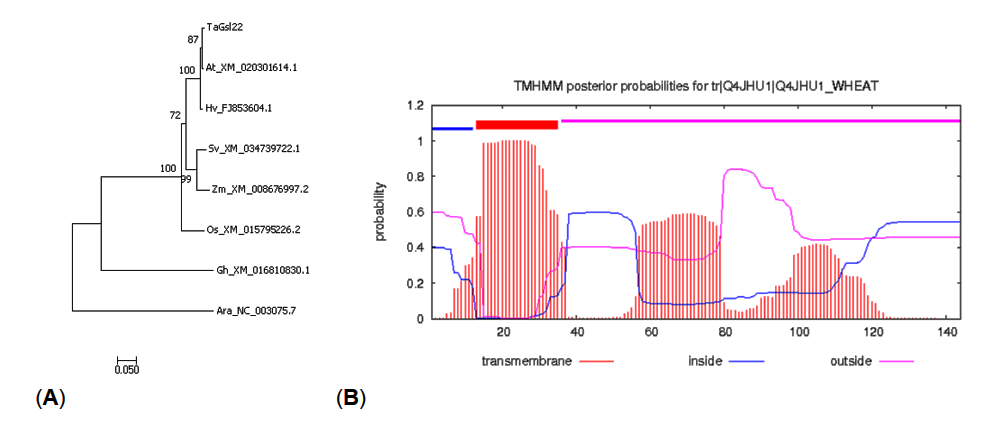
**

**Figure S1**. Bioinformatics analysis of *TaGSL22*. (**A**) Phylogenetic analysis of TaGSL22 protein and its orthologs in other plant species. Branches were labelled with the species names and GenBank accession numbers. Neighbor-Joining method, bootstraps = 1000, Ta: *Triticum aestivum*, At: *Aegilops tauschii*, Hv: *Hordeum vulgare*, Sv: *Setaria viridis*, Zm: *Zea mays*, Os: *Oryza sativa*, Gh: *Gossypium hirsutum*, Ara: *Arabidopsis thaliana*. (**B**) Transmembrane structure analysis of the protein encoded by *TaGSL22*.
